# Hippocampal subfield volumes in treatment resistant depression and serial ketamine treatment

**DOI:** 10.3389/fpsyt.2023.1227879

**Published:** 2023-10-09

**Authors:** Artemis Zavaliangos-Petropulu, Shawn M. McClintock, Shantanu H. Joshi, Brandon Taraku, Noor B. Al-Sharif, Randall T. Espinoza, Katherine L. Narr

**Affiliations:** ^1^Ahmanson-Lovelace Brain Mapping Center, Department of Neurology, Geffen School of Medicine at the University of California, Los Angeles, Los Angeles, CA, United States; ^2^Division of Psychology, Department of Psychiatry, UT Southwestern Medical Center, Dallas, TX, United States; ^3^Jane and Terry Semel Institute for Neuroscience and Human Behavior, Department of Psychiatry and Biobehavioral Sciences, Geffen School of Medicine at the University of California, Los Angeles, Los Angeles, CA, United States

**Keywords:** hippocampus, depression, ketamine, neurocognitive, treatment resistant, MRI

## Abstract

**Introduction:**

Subanesthetic ketamine is a rapidly acting antidepressant that has also been found to improve neurocognitive performance in adult patients with treatment resistant depression (TRD). Provisional evidence suggests that ketamine may induce change in hippocampal volume and that larger pre-treatment volumes might be related to positive clinical outcomes. Here, we examine the effects of serial ketamine treatment on hippocampal subfield volumes and relationships between pre-treatment subfield volumes and changes in depressive symptoms and neurocognitive performance.

**Methods:**

Patients with TRD (*N* = 66; 31M/35F; age = 39.5 ± 11.1 years) received four ketamine infusions (0.5 mg/kg) over 2 weeks. Structural MRI scans, the National Institutes of Health Toolbox (NIHT) Cognition Battery, and Hamilton Depression Rating Scale (HDRS) were collected at baseline, 24 h after the first and fourth ketamine infusion, and 5 weeks post-treatment. The same data was collected for 32 age and sex matched healthy controls (HC; 17M/15F; age = 35.03 ± 12.2 years) at one timepoint. Subfield (CA1/CA3/CA4/subiculum/molecular layer/GC-ML-DG) volumes corrected for whole hippocampal volume were compared across time, between treatment remitters/non-remitters, and patients and HCs using linear regression models. Relationships between pre-treatment subfield volumes and clinical and cognitive outcomes were also tested. All analyses included Bonferroni correction.

**Results:**

Patients had smaller pre-treatment left CA4 (*p* = 0.004) and GC.ML.DG (*p* = 0.004) volumes compared to HC, but subfield volumes remained stable following ketamine treatment (all *p* > 0.05). Pre-treatment or change in hippocampal subfield volumes over time showed no variation by remission status nor correlated with depressive symptoms (*p* > 0.05). Pre-treatment left CA4 was negatively correlated with improved processing speed after single (*p* = 0.0003) and serial ketamine infusion (*p* = 0.005). Left GC.ML.DG also negatively correlated with improved processing speed after single infusion (*p* = 0.001). Right pre-treatment CA3 positively correlated with changes in list sorting working memory at follow-up (*p* = 0.0007).

**Discussion:**

These results provide new evidence to suggest that hippocampal subfield volumes at baseline may present a biomarker for neurocognitive improvement following ketamine treatment in TRD. In contrast, pre-treatment subfield volumes and changes in subfield volumes showed negligible relationships with ketamine-related improvements in depressive symptoms.

## Introduction

About 30% of patients with major depressive disorder (MDD) suffer from symptoms that remain intractable despite two or more adequate antidepressant treatment trials (1), for which their MDD is defined as treatment resistant depression (TRD). In the last decade, ketamine, an N-methyl-D-aspartate receptor (NMDAR) antagonist, has emerged as a promising fast-acting treatment for TRD (2, 3). When administered at a subanesthetic dose, ketamine can produce a profound reduction of depressive symptom severity within hours in 40–60% of patients (4, 5). To advance more effective, fast-acting and low-risk antidepressant treatment modalities, much work has attempted to characterize the mechanisms underlying ketamine’s antidepressant effects.

Preclinical research suggests that part of the therapeutic effects of ketamine may be attributed to neuroplastic changes derived from improved neurotrophic signaling. The downstream effects of ketamine’s NMDAR antagonism triggers a number of signaling cascades, such as elevated glutamatergic firing and enhanced brain derived neurotrophic factor (BDNF) release (6, 7), leading to increased hippocampal spine density (8, 9). At the level of functional brain systems, ketamine-related neuroplasticity is hypothesized to relate to increased functional connectivity (10), cerebral blood flow (11) and glucose metabolism (12) observed in the hippocampus in patients with depression following ketamine treatment. Though studies are few and negative findings exist, some neuroimaging research suggests that ketamine treatment leads to increases in gross hippocampal volume (13, 14) and/or in CA4 and GC-ML-DG hippocampal subfields (14).

Research on conventional antidepressant treatments suggest that patients with larger pre-treatment hippocampal volume are more likely to have better treatment outcomes (15, 16). There is also some initial evidence that pre-treatment hippocampal volumes correlate with ketamine antidepressant treatment response. Specifically, larger right gross hippocampal volume (17) and left anterior subiculum volume (14) have been associated with antidepressant response following ketamine treatment, but those findings have had limited replication (18). Notably, these ketamine studies have primarily focused on changes in depressive symptoms as a measure of therapeutic response. While the hippocampus is involved in emotional processing (19), it plays a pivotal role in cognitive function, particularly memory (20). Emerging evidence suggests subanesthetic ketamine treatment improves neurocognitive function (21, 22) as well as depressive symptoms. Cognitive difficulties are a core symptom of depression that often persist after improvements in mood (23) and can impact quality of life and overall functional outcomes (24). To date, the relationship between pre-treatment hippocampal volumes and changes in neurocognition following ketamine treatment is largely unknown.

This study was designed to clarify the effects of ketamine treatment on change in hippocampal subfield volumes and its clinical and neurocognitive correlates in patients with TRD who received four intravenous serial ketamine treatments. First, we compared pre-treatment hippocampal subfields in patients to healthy controls and hypothesized that patients with TRD would have smaller hippocampal subfields compared to healthy controls. Next, we investigated changes in subfield volume in patients over the course of ketamine treatment and based on prior findings (13, 14) hypothesized that there would be significant increases in volume, most prominently in patients who achieved remission. We then tested for associations between pre-treatment subfield volume and change in clinical and neurocognitive outcomes, which to our knowledge have not been previously examined. We hypothesized that patients with larger pre-treatment subfield volumes would show greater improvements in depressive symptoms (14, 17) as well as in neurocognitive performance.

## Methods

### Participants

Patients who met DSM-5 diagnostic criteria for MDD (25) and experienced inadequate response to 2 or more prior antidepressant trials of sufficient dose and duration and had been continuously depressed for at least 6 months were recruited. Further details on inclusion and exclusion criteria have been previously described (22, 26, 27). Briefly, inclusion criteria entailed men and women between the ages of 20–64, pre-treatment moderate to severe depressive symptoms (17-item Hamilton Depression Rating Scale (HDRS) total score ≥ 17), stable antidepressant or mood stabilizer use for 6 or more weeks prior to study participation. Exclusion criteria entailed dementia diagnosis, patients experiencing a first major depressive episode, schizophrenia, neurological condition or serious medical illness, or substance abuse. Healthy controls (HC) group matched for age (within 2 years) and sex with no current or past psychiatric condition and no history of substance abuse or dependence were included. All participants provided written informed consent following procedures approved by the UCLA Institutional Review Board (IRB).

### Study design

**Figure 1:**
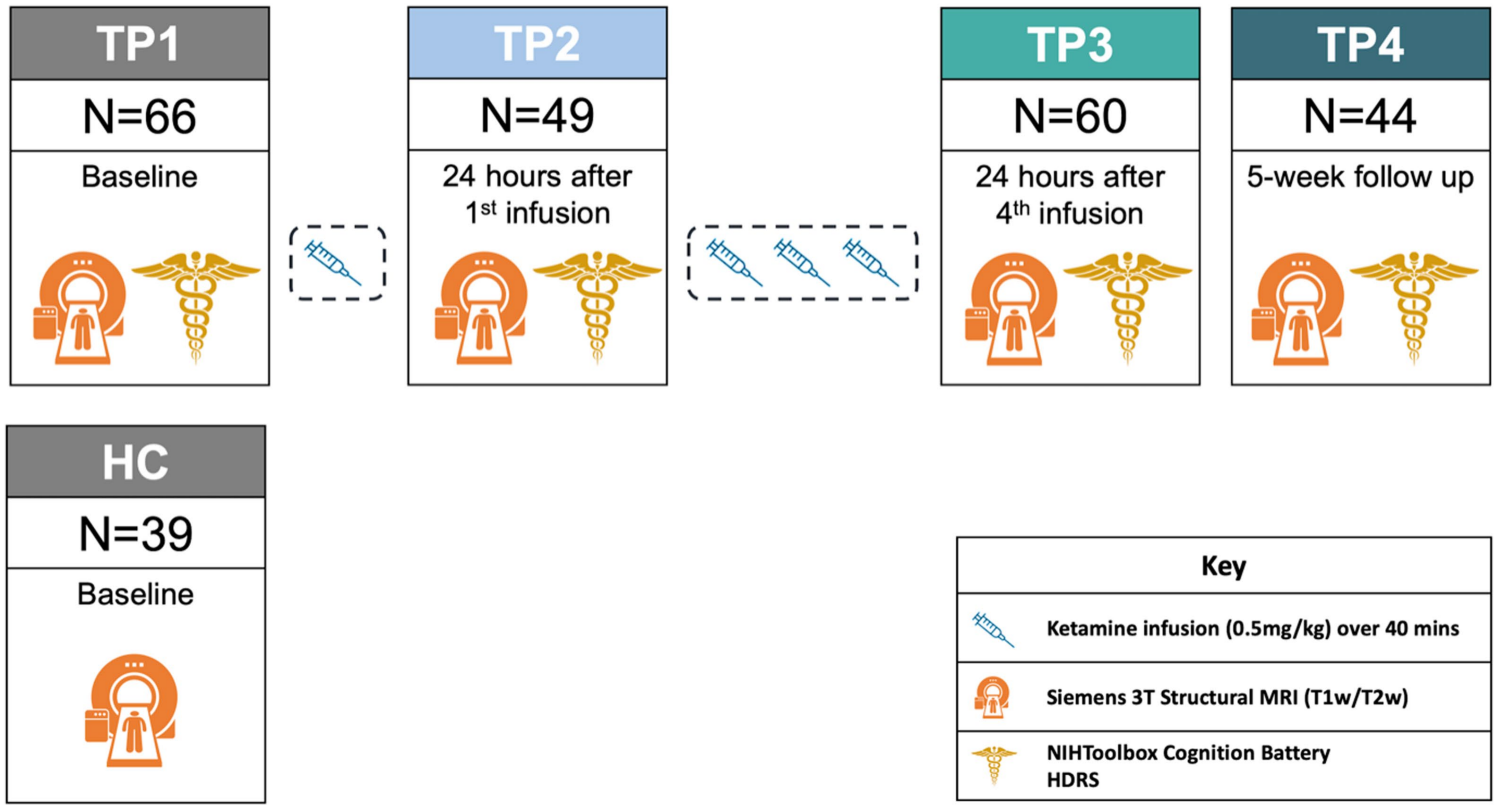
Ketamine study design. For demographic information about patients by time point, please refer to Supplementary Table S3.

Patients received open-label ketamine treatment 2–3 times a week for a total of 4 infusions over a 14-day period (NCT02165449). Clinical assessments and brain imaging scans for patients were collected pre-treatment (TP1), 24 h after the first infusion (TP2), 24 h after the fourth infusion (TP3), and 5 weeks following the end of treatment (TP4). HC brain imaging was collected at one time point. HC did not receive ketamine (Figure 1).

### Ketamine treatment

Patients received four serial infusions of racemic ketamine (0.5 mg/kg) administered intravenously over 40 min over the course of 2 weeks at the UCLA Clinical and Translational Research Center or Resnick Neuropsychiatric Hospital. Patients were permitted to remain on antidepressant medications so long as they were stable for at least 6 weeks prior to start of treatment. Benzodiazepines were discontinued throughout treatment. Details on concurrent antidepressant medication for patients can be found in Supplementary Table S1.

### Clinical assessments

Neurocognitive performance was assessed using the NIHToolbox Cognition Battery (28), which includes seven neurocognitive measures that assess specific cognitive domains. These domains include: Picture Vocabulary (probing language function), Flanker Inhibitory Control and Attention Test (probing attention and inhibition), List Sorting Working Memory Test (probing working memory), Dimensional Change Card Sort Test (probing executive function), Pattern Comparison Processing Speed Test (probing processing speed), Picture Sequence Memory Test (probing episodic memory), and Oral Reading Recognition Test (probing language). Composite scores for crystallized neurocognitive performance were calculated by averaging the scores from Oral Reading and Picture Vocabulary. Details regarding the seven neurocognitive assessments can be found in Supplementary Table S2. Composite scores for fluid neurocognitive performance were calculated by averaging the scores from the other five measures. Using the NIHToolbox guidelines, all scores (including composite scores) were adjusted for demographic factors (sex and age) and the resulting z-scores were analyzed.

Depressive symptoms were measured using the 17-item Hamilton Depression Rating Scale (HDRS) (29). Remitters were defined as patients with a HDRS total score of ≤7 (30, 31) 24 h after the fourth infusion.

### MRI acquisition

All participants were scanned at the UCLA Ahmanson-Lovelace Brain Mapping center on a Siemens 3T Prisma MRI System using a 32-channel head coil at each time point. Imaging sequences were performed according to the Human Connectome Project (HCP) Lifespan studies for Aging and Development (32). T1-weighted (T1w) multi-echo MPRAGE (0.8 mm isotropic; repetition time (TR) = 2500 ms; multi-echo time (TE) = 1.81:1.79:7.18 ms; inversion time (TI) = 1000 ms; flip angle (34) = 8.0°; acquisition time (TA) = 8:22 min) and T2-weighted (T2w; 0.8 mm isotropic; TR = 3200 ms; TE = 564 ms; TA = 6:35 min) were acquired with real-time motion correction (33). Both T1w and T2w images were acquired with a sagittal field-of-view of 256 × 240 × 166 mm with matrix size 320 × 300 × 208 slices, as described in (32).

### MRI data analysis

**Figure 2:**
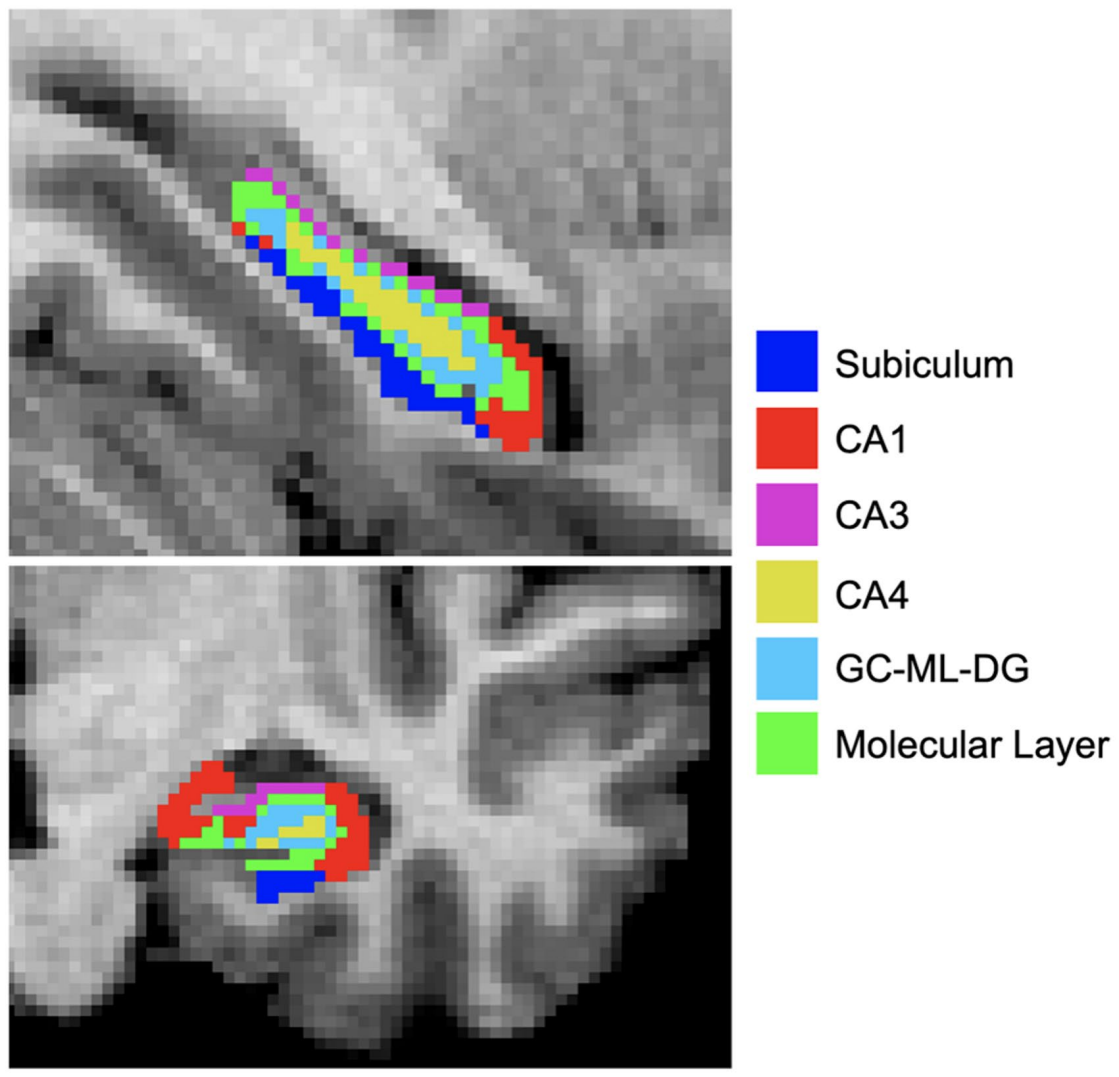
Hippocampal subfields were estimated using FreeSurferv7.2. Subfield segmentations are overlaid on a T1w image and color coded for visualization purposes. CA, Cornu ammonis; GC-ML-DG, Granule cell molecular layer of dentate gyrus.

T1w and T2w images were processed using the HCP minimal preprocessing pipeline (34). The longitudinal FreeSurfer v7.2 pipeline, which estimates whole hippocampal and subfields volumes was applied for segmentation, with both the T1w and T2w images at input for more reliable segmentation (35). For the purpose of this study, we investigated CA1, CA3, CA4, subiculum, molecular layer, and GC-ML-DG (Figure 2), merging head and body subcomponents as recommended by FreeSurfer.^1^ We did not analyze the hippocampal fissure, fimbria, HATA, and parasubiculum as these segmentations appear prone to measurement inaccuracies (36). Subfields were visually inspected for quality using the ENIGMA Hippocampal Subfield Quality Control protocol (37). Left and right subfields were analyzed separately after normalizing for whole hippocampal volume:


$$NormalizedSubfieldVolume=\frac{SubfieldVolume}{WholeHippocampalVolume}.$$

### Statistical analyses

All statistical analyses were conducted in R version 4.1.3. Hippocampal volumes were assessed at each time point for normality using density plots, q-q plots, and the Shapiro Wilk test. Each test was corrected for multiple comparisons using Bonferroni correction (0.05/6 subfields: *p* ≤ 0.008).

Linear regression models were used to compare normalized hippocampal subfield volumes between HC and TRD patients pre-treatment, correcting for age and sex (*Model: Hippocampal subfield ~ Diagnosis + age + sex*). A similar model was used to compare pre-treatment subfield volume between remitters and non-remitters, covarying for age, sex, and pre-treatment HDRS (*Model: Hippocampal subfield ~ Remission status + age + sex + pretreatment HDRS*). *Post-hoc* comparisons investigating patient-control differences in whole hippocampal volume (normalized for total intracranial volume) were also performed.

To assess change in hippocampal subfield volume occurring with treatment, linear mixed effects models (*nlme*) tested for the main effect of time, including the first three time points (TP1, TP2, and TP3) for each hippocampal subfield (*Model: Hippocampal subfield ~ time + random(participant)*). To test for potential differences in change in subfield volume between remitters and non-remitters, remission was included as an interaction term (Model: Hippocampal subfield ~ time*Remission status + random(participant)). *Post-hoc* comparisons investigated time points pairwise, including the final follow-up time point (TP4). Additional *post-hoc* comparisons of change in whole hippocampal volume *(normalized for total intracranial volume)* were also performed.

Linear regression models also tested for associations between normalized pretreatment subfield volume and percent change in HDRS and NIHToolbox measures, adjusting for age and sex. As a follow up, a linear regression model was used to test for an interaction between pretreatment subfield volume and remission status in association with percent change in neurocognitive performance after TP3 ((TP3-TP1)/TP1). Age, sex, and baseline HDRS were included in the model as covariates (Model: Neurocognitive score ~ Pretreatment subfield*Remission status + age + sex + pretreatment HDRS).

## Results

### Participants

Means and standard deviations for demographic characteristics of patients and HC are provided in Table 1. Patients showed highly significant improvements in HDRS total scores between baseline and end of serial ketamine treatment (*t* = 13.0, *p* < 0.001). Clinical improvements diminished by five-week follow-up, though remained significantly lower compared to baseline (*t* = 8.2, *p* < 0.001). As recently reported by our group (22), patients showed significant improvements in neurocognitive performance following ketamine treatment. Specifically, patients showed significant improvements (*p* < 0.001) in composite fluid neurocognitive performance, flanker inhibition, and processing speed between baseline and end of serial ketamine treatment that was sustained through the 5-week follow-up time period. We have previously reported on the significant increases in neurocognitive performance and depressive symptoms in this dataset (22).

**Table 1:** Participant demographics.

	TRD	Healthy controls	*t*/*χ*^2^	*p*
*N*	66	32	–	–
Age (years)	39.5 ± 11.1	35.03 ± 12.2	−1.8	0.08
Sex	31M/35F	17M/32F	0.33	0.56
Race – White, Non-Hispanic	66.6%	26%	–	–
Race – White, Hispanic	10.1%	17.9%	–	–
Race – Asian	10.1%	17.9%	–	–
Race – Black	3%	20.5%	–	–
Race – More than 1	3%	7.7%	–	–
Duration of lifetime illness (years)	24.5 ± 15.5	–	–	–
Age of onset (years old)	16.6 ± 8.4	–	–	–
Pre-treatment HDRS	19 ± 4.9	–	–	–
Pre-treatment composite crystallized neurocognitive function	57.3 ± 9.4	–	–	–
Pre-treatment composite fluid neurocognitive function	47.3 ± 9.2	–	–	–

### Patients vs. healthy controls

**Figure 3:**
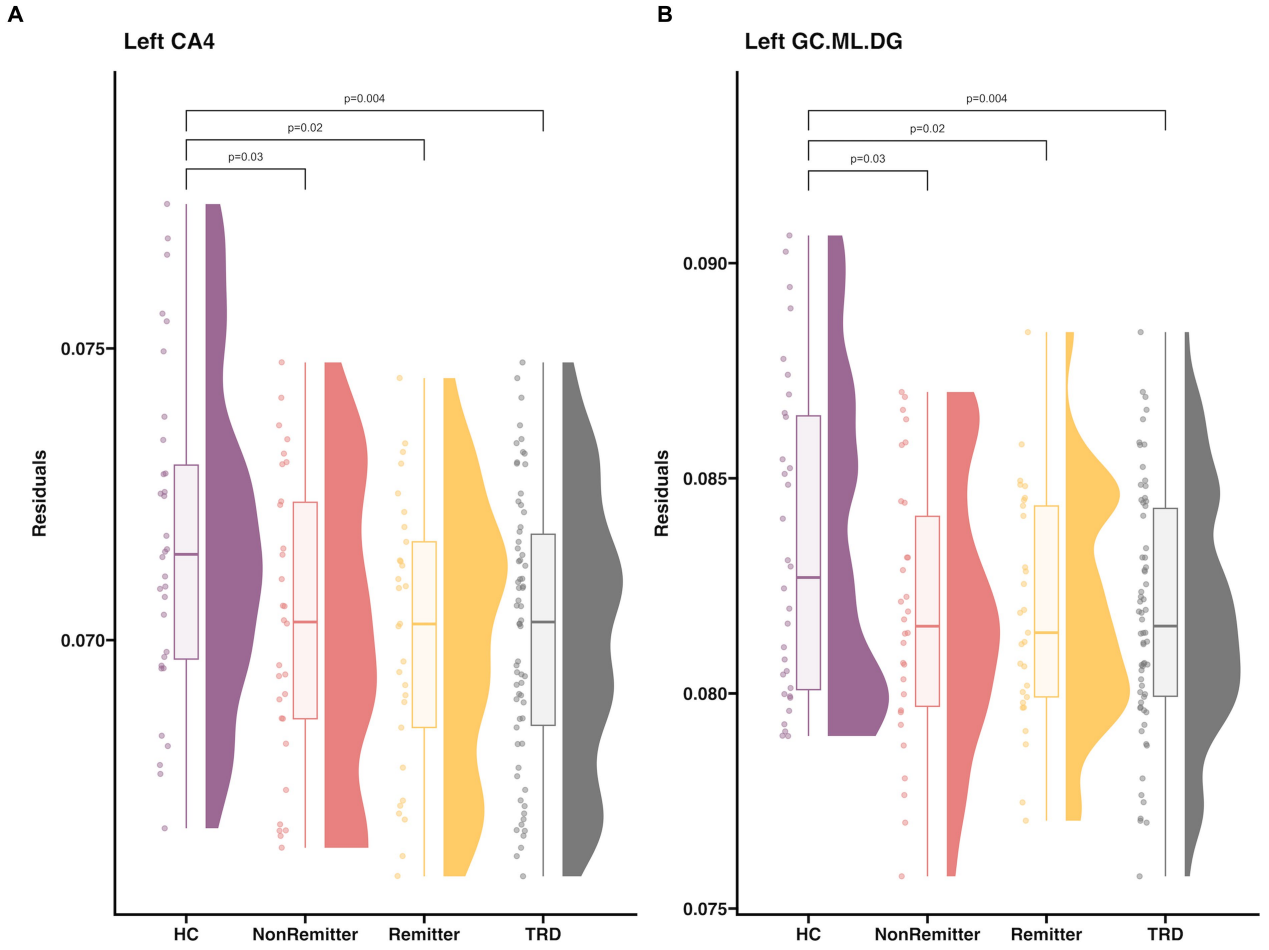
Raincloud plot that includes HC, baseline TRD, Remitters, NonRemitters for **(A)** left CA4 and **(B)** left GC.ML.DG. Subfield volume (*y*-axis) is residualized for age and sex. Plots and descriptive statistics for group comparisons can be found in Supplementary Tables S4, S5 and Supplementary Figures S1, S2. A summary of all hippocampal subfields can be found in Supplementary Figure S3. Twenty-nine patients achieved remission (HDRS≤7, age = 43.3 ± 11.9, 13M/16F) and 30 patients did not achieve remission (age = 37.1 ± 9.9, 16M/14F).

Pre-treatment, patients with TRD had smaller left CA4 (*d* = −0.6, *t* = −2.9, *p* = 0.004) and GC.ML.DG volumes compared to HC (*d* = −0.61, *t* = −2.95, *p* = 0.004) (Figure 3). No significant differences in subfield volumes between HC and TRD were observed in the right hemisphere or in post-hoc whole hippocampal volume in either hemisphere (*p* > 0.05).

### Change in subfield volumes throughout treatment

Linear mixed effect models showed no significant change in volume over time for any subfield (*p* > 0.05, Supplementary Table S6 and Supplementary Figure S3). *Post-hoc t*-tests revealed trend-level increases in left hippocampus from TP1 to TP4 (5-weeks post treatment), specifically CA3 (*d* = 0.65, *t* = 2.12, *p* = 0.03), CA4 (*d* = 0.63, *t* = 2.04, *p* = 0.05), and GC.ML.DG (*d* = 0.63, *t* = 2.05, *p* = 0.05) (Supplementary Figure S3). Furthermore, there was no significant interaction between remitter status and change in volume for any subfield (*p* > 0.05). Further *post-hoc* analysis showed no change in whole hippocampal volume in either hemisphere (*p* > 0.05).

### Pre-treatment subfield volume associations with changes in depressive symptoms

There were no significant associations between pre-treatment subfield volumes and changes in HDRS at any time point (*p* > 0.05). Also, there were no significant differences in pre-treatment subfield volumes between remitters and non-remitters (*p* > 0.05, Figure 3).

### Pre-treatment subfield volume associations with changes in neurocognitive performance

#### Processing speed

**Figure 4:**
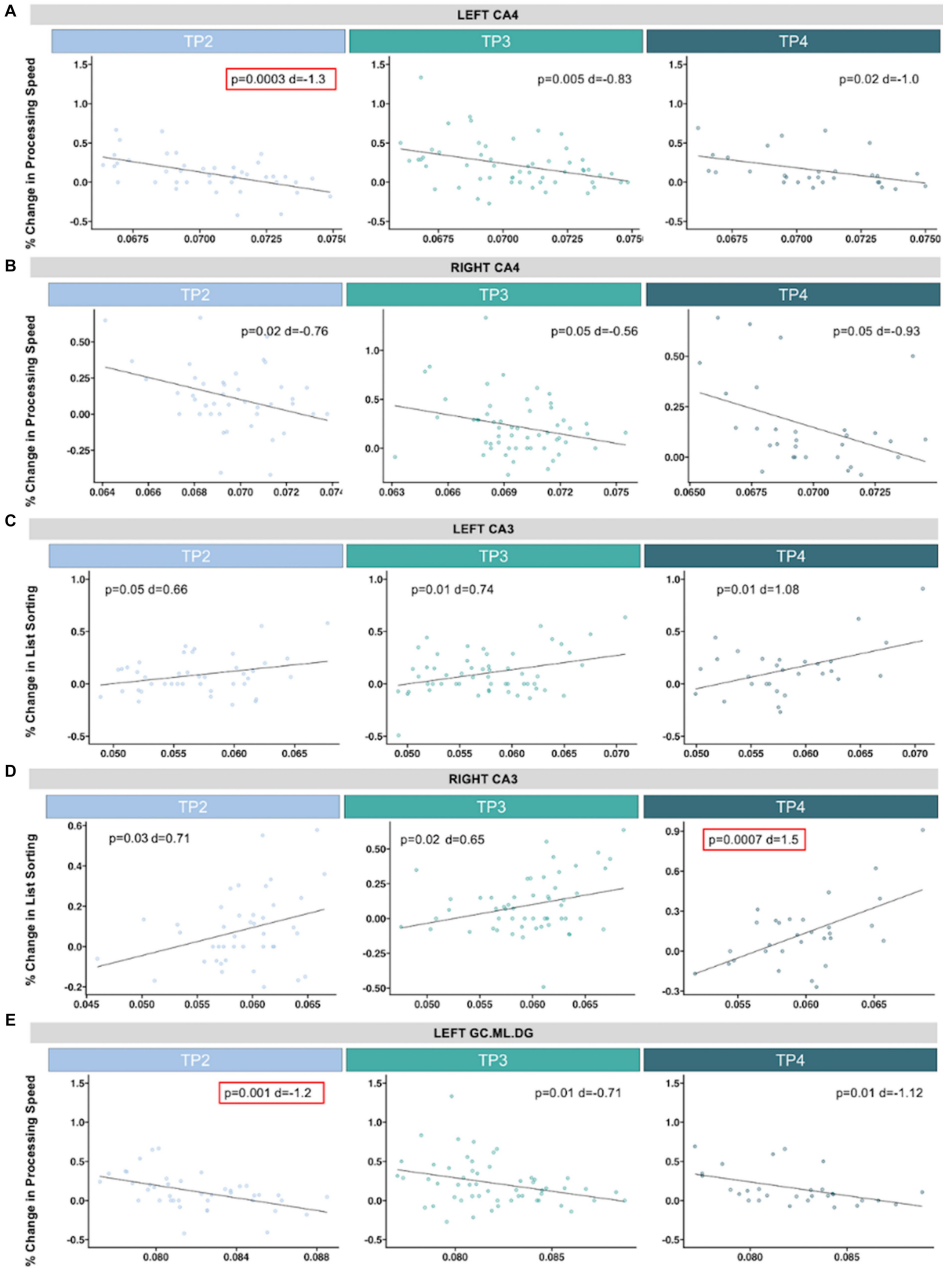
Scatterplots show associations between pre-treatment hippocampal volume (*x*-axis, residualized for age and sex) and percent change in neurocognitive performance (*y*-axis). Significant associations that passed correction for multiple comparisons are outlined in red. In the CA4, we identified a significant association between greater improvements in processing speed and smaller **(A)** left pre-treatment hippocampal volume and 24 h after single ketamine infusion, with trending associations (*p* < 0.05) after serial infusion and follow up, and **(B)** across time points in the right hemisphere. **(C)** Larger pre-treatment right CA3 was significantly associated with improvements in list sorting (an assessment of working memory) at follow-up. **(D)** Similar trending associations were observed at the other time points and in the left hemisphere. **(E)** Improvements in processing was also significantly associated with smaller pre-treatment left GC.ML.DG after single infusion, with trending associations for the remaining time points. Descriptive statistics for associations between pre-treatment hippocampal subfields and neurocognitive assessments across timepoints can be found in Supplementary Tables S7–S9.

Pre-treatment left CA4 was negatively associated with improvements in processing speed after single (TP2: *p* = 0.0003, *d* = −1.3, *t* = −4.02) and serial ketamine treatments (TP3: *p* = 0.005, *d* = −0.83, *t* = −2.96) (Figure 4). Similar trends toward significance were observed with processing speed in the left CA4 at follow-up (TP4: *p* = 0.02, *d* = −1.0, *t* = −2.54) and in right CA4 after each time point (TP2: *p* = 0.02, *d* = −0.76, *t* = −2.34; TP3: *p* = 0.05, *d* = −0.56, *t* = −2.0; TP4: *p* = 0.05, *d* = −0.93, *t* = −2.0), but did not pass Bonferroni correction. Pre-treatment left GC.ML.DG was also negatively correlated with changes in processing speed after single infusion (TP2: *p* = 0.001, *d* = −1.2, *t* = −3.53), with similar trends in the left hemisphere after the fourth infusion (TP3: *p* = 0.01, *d* = −0.71, *t* = −2.53) and follow-up (TP4: *p* = 0.01, *d* = −1.12, *t* = −2.85) and the right hemisphere after fourth infusion (TP4: *p* = 0.03, *d* = −0.91, *t* = −2.33).

#### List sorting working memory

Right pre-treatment CA3 was positively associated with changes in list sorting working memory at follow-up (TP4: *p* = 0.0007, *d* = 1.5, *t* = 3.8), with similar trends at TP2 (*p* = 0.03, *d* = 0.71, *t* = 2.20) and TP3 (*p* = 0.02, *d* = 0.65, *t* = 2.32) (Figure 4). Left CA3 also showed trends of positive associations with list sorting working memory (TP2: *p* = 0.05, *d* = 0.66, *t* = 2.03; TP3: *p* = 0.01, *d* = 0.74, *t* = 2.66; TP4: *p* = 0.01, *d* = 1.08, *t* = 2.69).

## Discussion

In this study, we sought to investigate the effects of serial ketamine treatment on hippocampal subfield volumes in patients with TRD and the relationships between pre-treatment subfield volumes and changes in both depressive symptoms and neurocognitive performance. In accordance with prior findings from our group and others, patients with TRD that were treated with serial ketamine showed highly significant reductions in depressive symptoms (4, 11, 38, 39) and also significant improvements in neurocognitive performance (21, 22, 40). Importantly, this current study provided new evidence that hippocampal subfield volume, including CA3, CA4, and GC.ML.DG, prior to ketamine treatment was associated with significant improvements in neurocognitive performance, specifically processing speed and working memory. In contrast, we failed to replicate prior findings that suggested that pre-treatment hippocampal volumes were related to improvements in depressive symptoms (17, 41). We also were unable to replicate prior findings that identified significant changes in hippocampal volume following ketamine treatment (13, 14, 41). Ketamine treatment remitter status did not impact these negative findings. However, increases in left CA3, CA4, and GC.ML.DG volumes from baseline to 5-week follow up trended toward significance, suggesting possible longer-term effects of ketamine on hippocampal structure. Finally, consistent with prior findings in the field suggested that depression was associated with smaller hippocampal volume (42, 43), we found that patients with TRD relative to HC had smaller left CA4 and GC.ML.DG baseline volumes.

The hippocampus is widely implicated in mood disorders given its critical role in limbic system function (44). Subfields within the anterior hippocampus, including CA3 and CA4/dentate, have been shown to be involved in pattern separation of emotionally charged stimuli (20), with fMRI research reporting activation in the anterior hippocampus in response to emotionally charged faces (45). Hippocampal dysfunction in MDD, including reduced volume (42, 43, 46–48) and aberrant activity patterns (49, 50), is suggested to contribute to both mood and neurocognitive symptoms. In this study, we found that patients with TRD had significantly smaller pre-treatment volumes in the left CA4 and GC.ML.DG when compared to HC. Reduced volumes of CA1 (43, 46), CA3 (42, 43, 46), CA4 (42, 43), and GC.ML.DG (42), have been reported in patients with MDD when compared to healthy controls, although the laterality of reports vary. There are several theories that speculate about the underlying biological mechanism driving reduced hippocampal volume observed in patients with depression (51). Amongst the more commonly proposed hypotheses is that reduced hippocampal volume in depression may be linked with impaired hippocampal adult neurogenesis (52) and/or be attributed to chronically hyperactive hypothalamic–pituitary–adrenal axis that leads to glucocorticoid neurotoxicity (53, 54). Given the number of glucocorticoid receptors in the hippocampus, the hippocampus is preferentially targeted, resulting in the observed hippocampal atrophy (44). Interestingly, increases in hippocampal volume have been observed following antidepressant treatment, which suggests a possible reversal of this process. (55) found increases in left CA3 and GC.ML.DG volumes following an 8-week study of various antidepressant medication. Increases in whole hippocampal volume and CA4 specifically have also been observed following electroconvulsive therapy (56–59), although the laterality of findings have been mixed.

We were unable to detect significant changes in hippocampal subfield volumes following single or serial ketamine infusions, even when considering remitter status. Given the small effect sizes (average effect size for the interaction between time and remission status across subfields: *F* = 0.046), our findings suggest that hippocampal volume change may have limited utility as an antidepressant response biomarker. We did, however, observe trends that suggested an increase in the left CA3, CA4, and GC.ML.DG from baseline to 5-week follow-up. Previous studies have investigated changes in hippocampal volume following antidepressant ketamine treatment (13, 14). Interestingly, (14) found significant increases in the left CA4 and GC.ML.DG 24 h following a 6th serial ketamine infusion. The (14) study used a two-week treatment course of 6 ketamine infusions, while our current study administered only 4 ketamine infusions over a similar period of time. It is possible that the two additional ketamine infusions administered in Zhou et al. may have accelerated the trending increase in left CA4 and GC.ML.DG that we observed at the 5-week follow-up. Abdallah et al. (13) found an increase in left whole hippocampal volume following treatment only in patients who remitted 24 h following a single ketamine infusion, a finding which was not replicated in the current study. Design differences may explain our failure to replicate this finding. For example, (13) defined treatment remission as patients with a total score of less than 10 on the Montgomery Åsberg Depression Rating Scale, a different measure from the HDRS that was used in the current study, although the two measures are highly correlated (60). However, the small sample size in (13) (*N* = 16) may best explain our failure to replicate that finding. Few studies have investigated how ketamine impacts brain structure, most likely due to the assumption that structural plasticity occurs slowly and is most likely not evident in the short follow-up design of most ketamine study designs (61). Further investigations over longer time periods of observation are necessary to understand how ketamine may impact hippocampal volume. While the current antidepressant ketamine literature suggests that it is possible that ketamine may increase hippocampal volume, it is important to ensure that hippocampal atrophy observed in ketamine abuse research (62) does not occur with repeated antidepressant ketamine treatments.

Some prior studies suggested a relationship between pre-ketamine treatment hippocampal volume and subsequent clinical response. Studies of gross hippocampal volume have reported larger pre-treatment volumes associated with significant improvements in depressive symptoms following a prolonged ketamine infusion treatment (17) and standard single ketamine infusion (63). Only one prior study has investigated subfields specifically and found larger pretreatment subiculum volumes in serial ketamine responders (41). Further, some evidence has suggested that hippocampal structure may serve as a useful biomarker of other antidepressant treatment efficacy (64–66). In our study, we failed to replicate these findings, as we observed no significant associations between pre-treatment hippocampal subfield volumes with change in depressive symptoms or remission status. Our work more closely replicates a separate ketamine study that reported null findings for associations between pre-treatment hippocampal volume and changes in depressive symptoms (18), ultimately suggesting that pre-treatment hippocampal volume is an unreliable biomarker of antidepressant response.

We did, however, identify significant associations with pre-treatment hippocampal subfield volumes and improvements in neurocognitive function. Smaller pre-treatment CA4 and GC.ML.DG were significantly associated with improvements in processing speed, most prominently after single ketamine infusion in the left hemisphere, but with trending associations across time points for both subfields and in the right hemisphere for CA4. Larger pre-treatment CA3 volumes were significantly associated with changes in list sorting working memory, an assessment of verbal and visual working memory, most strongly at follow-up and in the right hemisphere, but trending associations were identified in both hemispheres and across time points. Further research investigating pre-treatment hippocampal subfields that look at inflammatory biomarkers, gene expression, or other biological markers is necessary to explain why larger pre-treatment CA3 and smaller CA4 and GC.ML.DG significantly associated with improvements in neurocognitive performance. It is possible that we identified associations between pre-treatment hippocampal subfields and changes in neurocognitive performance, but not depressive symptoms because neurocognitive performance relative to emotional function is more closely linked with hippocampal subfields. Although, as previously discussed, patients with depression have shown smaller hippocampal subfields than healthy controls (43), and mood symptoms were found to be unassociated with hippocampal volume (47, 67). However, some studies have successfully mapped neurocognitive performance to hippocampal subfield volumes in cognitively healthy populations (68) and populations with cognitive impairment (68–70). Despite mixed findings in prior studies, it is possible that associations with pre-treatment hippocampal volumes may be influenced by concurrent antidepressant medication (71, 72). For example, lithium has been found to associate with larger hippocampal volume in patients with bipolar disorder (73). Though one participant reported concurrent lithium use in this study, results remained consistent with and without inclusion of this participant in analysis. Additionally, the results remained stable when including a covariate regarding medication status (medicated vs. unmedicated), despite the limited sample of unmedicated participants (*n* = 12 unmedicated). Notably, for this study, medication was required to have remained stable for 6 weeks prior to and during treatment, however we were not statistically powered to examine the effects of individual medications (Supplementary Table S1). Thus it remains possible that medication status could contribute to observations of larger pre-treatment CA3 and smaller pre-treatment CA4 and GC.ML.DG associations with improvements in neurocognitive performance. The potential pro-cognitive effects of antidepressant ketamine treatment have only been recently explored (21, 22). To our knowledge, this is the first study to report associations between pre-treatment hippocampal subfield volumes and the pro-cognitive effects of antidepressant ketamine treatment, therefore, further replication is necessary to determine the generalizability of these findings.

## Limitations

The are several limitations to consider for contextualizing the reported findings. The study was an open-label naturalistic design and had no placebo control group. The object of the study was to model biologically changes following antidepressant ketamine treatment, rather than to determine the antidepressant efficacy of ketamine treatment (26, 74). Furthermore, patients were not primed to anticipate any improvements in neurocognitive performance. Given the lack of placebo, we are unable to account for the influence of pre-treatment hippocampal volume.

Additionally, while the NIH Toolbox can be used for longitudinal assessments, practice effects may still occur with repeated testing (28). We have previously examined practice effects in the NIHToolbox measures used in this study in an overlapping sample of patients treated with ketamine (22) and a small sample of healthy controls assessed twice at similar intervals across time. Though both patients and controls showed improvements in the flanker inhibition task and processing speed, patients showed significantly greater change than controls. In a supplemental post-hoc analysis of relationships between baseline hippocampal volume in this sample of controls, we found no relationships with change in neurocognitive performance. This suggests that pre-treatment hippocampal volume likely does not predict practice effects or predispose to improved cognition over time. However, further investigation of changes in neurocognitive performance following ketamine treatment with more sensitive assessments and placebo control is necessary.

Patients were permitted to remain on concurrent antidepressant medication, so long as it was stable for at least 6 weeks prior. The distribution of concurrent medication in the current sample was insufficient to investigate the potential confounding effects on hippocampal subfields for this study, but are reported in Supplementary Table S1. Finally, ultra-high resolution scans acquired on a 7 Tesla MRI is optimal for delineating the subtle boundaries of hippocampal subfields. Although the current study was acquired on a 3T MRI, we employed validated methods that utilize both T1 and T2 images, which has been shown to improve segmentation accuracy (35). Furthermore, we excluded smaller, less reliable segmentations from the analysis.

## Conclusion

To our knowledge, in this study we reported for the first-time new results that suggest pre-treatment hippocampal subfield volume may present a useful biomarker for pro-cognitive effects of antidepressant ketamine treatment. Further replication of this new finding is necessary to confirm these results and determine the generalizability and clinical relevance. In contrast, pre-treatment hippocampal subfield volumes showed negligible relationships with ketamine-related improvements in mood symptoms and subfield volumes did not significantly change over time.

## Data availability statement

The raw data supporting the conclusions of this article will be made available by the authors, without undue reservation.

## Ethics statement

The studies involving humans were approved by University of California Los Angeles Institutional Review Board. The studies were conducted in accordance with the local legislation and institutional requirements. The participants provided their written informed consent to participate in this study.

## Author contributions

AZ-P, SM, and KN: study conception and design. BT, NA-S, RE, and KN: data collection. AZ-P, SM, KN, SJ, NA-S, and BT: analysis and interpretation of results. AZ-P, SM, SJ, RE, and KN: draft manuscript preparation. All authors reviewed the results and approved the final version of the manuscript.

## Footnotes

^1^ https://surfer.nmr.mgh.harvard.edu/fswiki/HippocampalSubfieldsAndNucleiOfAmygdala
