# Erratum: Hippocampal subfield volumes in treatment resistant depression and serial ketamine treatment

**DOI:** 10.3389/fpsyt.2024.1371092

**Published:** 2024-01-30

**Authors:** Frontiers Production Office

**Affiliations:** Frontiers Media SA, Lausanne, Switzerland

**Keywords:** Hippocampus, depression, ketamine, neurocognitive, treatment resistant, MRI

Due to an editorial error, the wrong reviewer affiliation was included for Yael Jacob. The correct affiliation is as follows:

Icahn School of Medicine at Mount Sinai, United States

The publisher apologizes for this mistake. The original version of this article has been updated.

