# Supplementary material for: Hippocampal subfield volumes in treatment resistant depression and serial ketamine treatment
Source: Front Psychiatry. 2023 Oct 9;14:1227879. doi: 10.3389/fpsyt.2023.1227879 (PMC10590913; doi:10.3389/fpsyt.2023.1227879)
Supplement: Supplementary file 1 [file Data_Sheet_1.docx]

# Supplemental Material


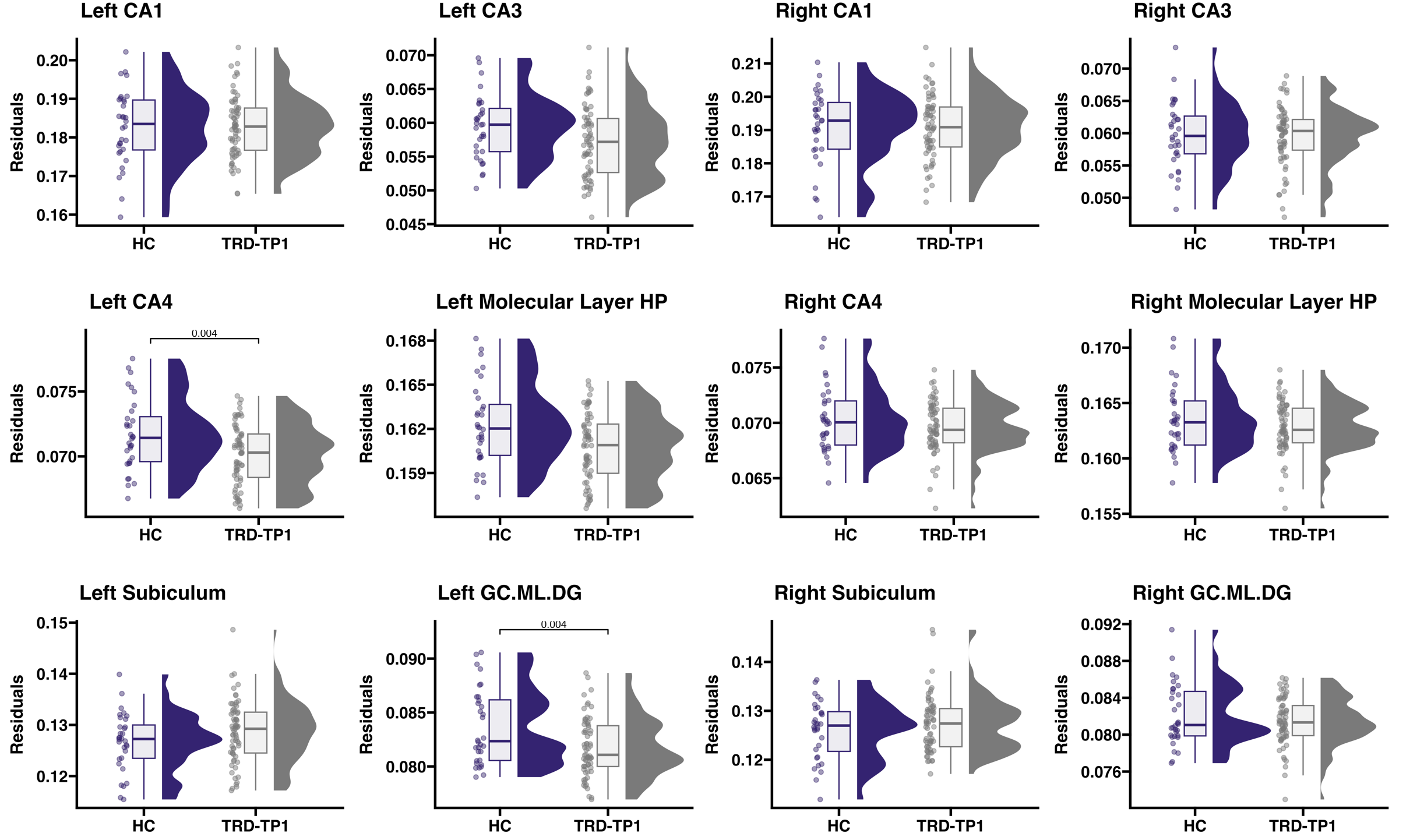


**Supplemental Figure 1.** Raincloud plots comparing hippocampal subfield volumes in healthy controls (HC) and in patients prior to treatment (TRD-TP1). Subfield volumes (y-axis) are residualized for age and

sex.


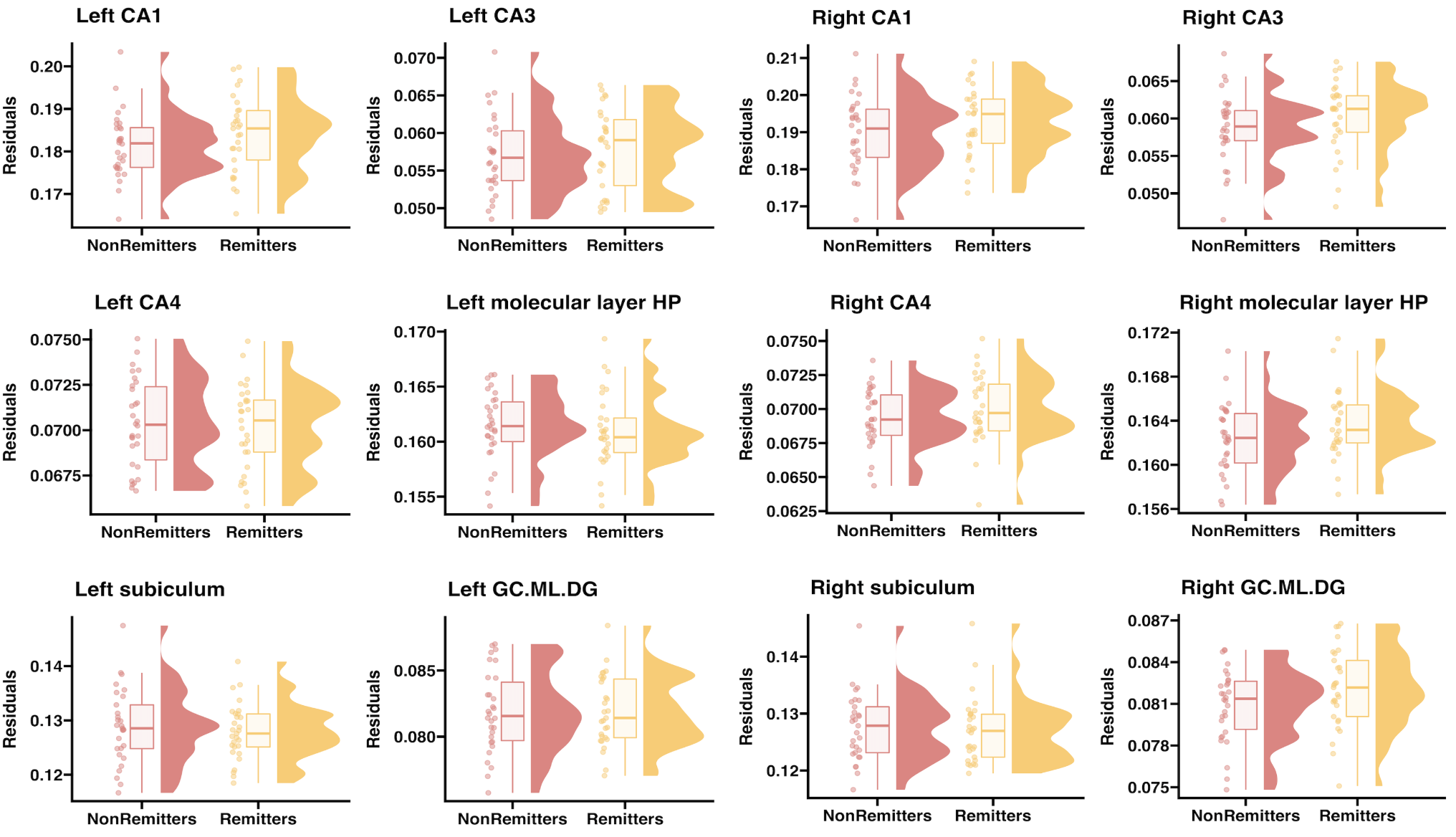


**Supplemental Figure 2.** Raincloud plots comparing pre-treatment hippocampal subfield volumes in remitters and non-remitters, as determined by their remission status at TP3 (post-fourth infusion). Subfield volumes (y-axis) are residualized for age, sex, and baseline HDRS.


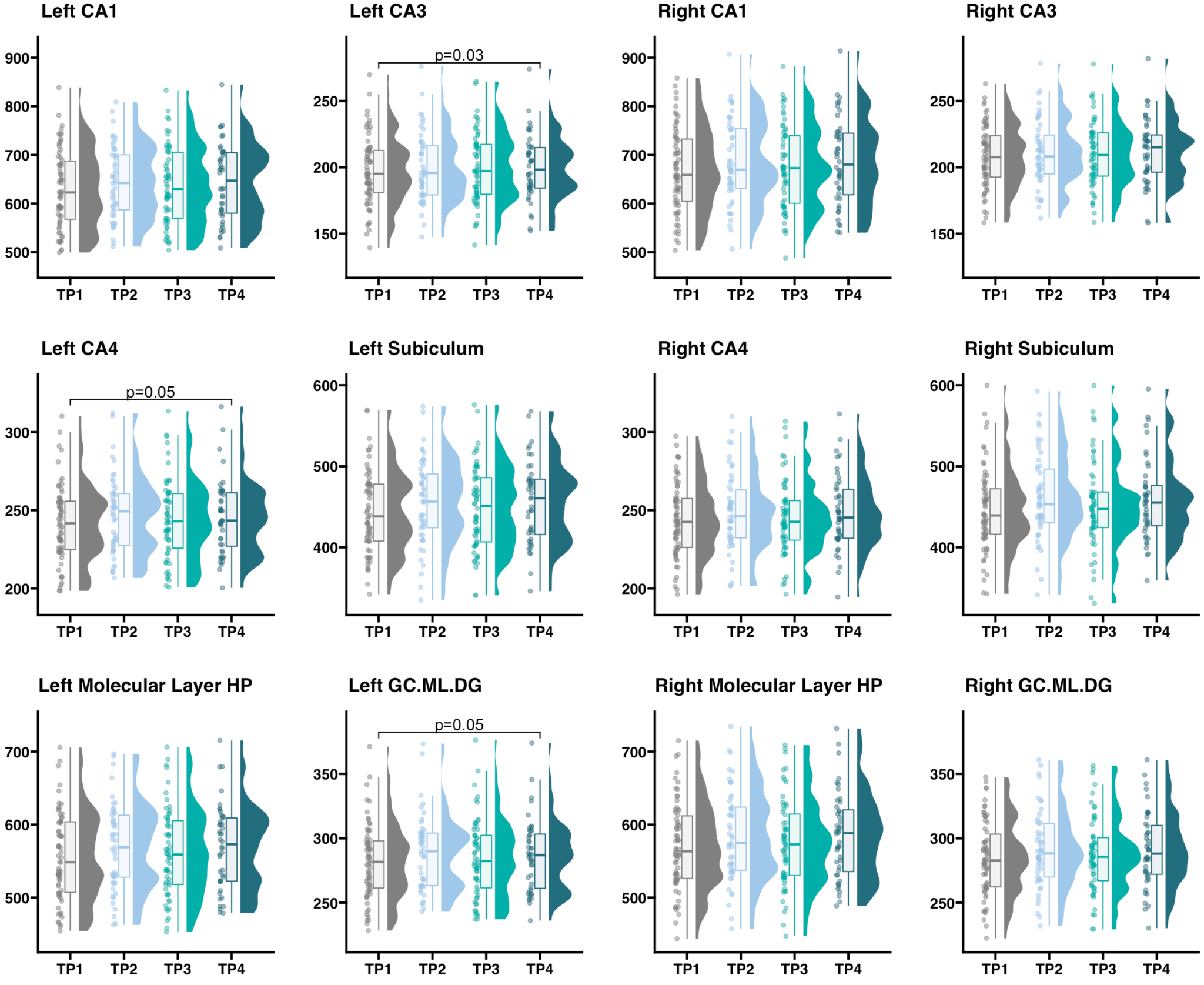


**Supplemental Figure 3.** Raincloud plots for hippocampal subfield volume over the course of treatment are presented. Moderate increases (p<0.05) were observed from pre-treatment to follow-up in the left CA3 and GC.ML.DG, but did not pass corrections for multiple comparisons.

**Supplemental Table 1.** Medication information for TRD participants.

| **Medication** | **Percentage** |
| --- | --- |
| Selective serotonin reuptake inhibitor | 27.3% |
| Serotonin-norepinephrine reuptake inhibitor | 28.8% |
| Other antidepressants | 50% |
| MAOIs | 3% |
| Lithium | 1.5% |
| Benzodiazepines (discontinued during treatment) | 27.3% |
| Anticonvulsants | 24.2% |
| Atypical Antipsychotics | 19.7% |
| Stimulants | 22.7% |

**Supplemental Table 2.** Detailed descriptions of the NIHToolbox Cognitive Battery Measures

| **Construct** | **Measure** | **Description** | **Test Time** |
| --- | --- | --- | --- |
| Language | Picture Vocabulary | Measures receptive vocabulary. Patients select the picture that most closely matches the meaning of the word | 4 minutes |
| Attention & Executive Function | Flanker Inhibitory Control and Attention | Measures the patient’s attention and inhibitory control. Assesses the limited capacity to deal with an abundance of environmental stimulation. | 3 minutes |
| Working Memory | List Sorting Working Memory | Evaluates immediate recall and the ability to store information until the amount of information to be stored exceeds one’s capacity to hold that information | 7 minutes |
| Executive Function | Dimensional Change Card and Sort | Evaluates cognitive flexibility. Assesses the ability to plan, organize and monitor executive behaviors that are specifically directed in a goal-oriented manner | 4 minutes |
| Processing Speed | Pattern Comparison Processing Speed Test | Assess how much information can be processed within a period of time | 3 minutes |
| Episodic Memory | Picture Sequence Memory | Measures cognitive processes involved in the acquisition, storage and retrieval of new information | 7 minutes |
| Language | Oral Reading Recognition Test | Evaluates reading decoding skill and crystallized abilities. Patients are asked to read and pronounce letters and words as precisely as possible | 3 minutes |

**Supplemental Table 3.** Demographics and raw whole hippocampus and hippocampal subfields volumes are listed by participants included at each time point.

|  | **TP1** | **TP2** | **TP3** | **TP4** |
| --- | --- | --- | --- | --- |
| **N** | 66 | 49 | 60 | 44 |
| **Age (years)** | 39.5±11.1 | 38.9±10.8 | 40.1±11.2 | 39.1±10.3 |
| **Sex** | 31M/35F | 28M/21F | 30M/30F | 24M/20F |
| **HDRS** | 19±4.9 | 12.7±4.6 | 8.3±4.6 | 11.2±5.6 |
| **Average whole hippocampal volume (mm^3^)** | 3445.3±342.7 | 3526.5±346.9 | 3481.3±346.1 | 3526.4±336.5 |
| **Average CA1 volume (mm^3^)** | 630.2±79.9 | 644.4±73.9 | 638.1±78 | 644.1±78.3 |
| **Average CA3 volume (mm^3^)** | 197.4±26.0 | 199.5±26.2 | 199.6±26.4 | 199.8±24.4 |
| **Average CA4 volume (mm^3^)** | 241.9±24.4 | 247.2±25.0 | 244.4±25.7 | 245.6±24.1 |
| **Average subiculum volume (mm^3^)** | 443.8±50.8 | 455.9±52.6 | 448.4±51.6 | 455.9±50.7 |
| **Average molecular layer hp volume (mm^3^)** | 555.7±60.8 | 569.1±59.2 | 561.7±60.5 | 569.0±57.9 |
| **Average GC.ML.DG volume (mm^3^)** | 281.9±29.1 | 288.4±29.1 | 248.9±30.1 | 286.8±28.1 |

**Supplemental Table 4.** Descriptive statistics from linear models comparing subfield volumes in patients with TRD and healthy controls.

|  | **Left** | | | | | **Right** | | | | |
| --- | --- | --- | --- | --- | --- | --- | --- | --- | --- | --- |
| **ROI** | **estimate** | **stderror** | **tval** | **dval** | **pval** | **estimate** | **stderror** | **tval** | **dval** | **pval** |
| CA1 | 0.0002 | 0.002 | 0.11 | 0.02 | 0.91 | 0.0001 | 0.002 | 0.06 | 0.01 | 0.95 |
| CA3 | -0.002 | 0.001 | -1.97 | -0.41 | 0.05 | -0.0002 | 0.001 | -0.23 | -0.5 | 0.82 |
| CA4 | -0.002 | 0.0005 | -2.9 | -0.60 | **0.004** | -0.0006 | 0.0006 | -1.15 | -0.24 | 0.25 |
| subiculum | 0.002 | 0.001 | 1.51 | 0.31 | 0.14 | 0.001 | 0.001 | 1.07 | 0.22 | 0.29 |
| molecular_layer_HP | 0.0001 | 0.0008 | 0.14 | 0.03 | 0.89 | -0.0007 | 0.0006 | -1.13 | -0.24 | 0.3 |
| GC.ML.DG | -0.002 | 0.006 | -2.95 | -0.61 | **0.004** | -0.0007 | 0.0006 | -1.14 | -0.24 | 0.25 |

**Supplemental Table 5.** Descriptive statistics from linear models comparing pre-treatment subfield volumes in remitters and non-remitters, as determined by their TP3 remission status.

|  | **Left** | | | | **Right** | | | |
| --- | --- | --- | --- | --- | --- | --- | --- | --- |
| **ROI** | **estimate** | **stderror** | **tval** | **pval** | **estimate** | **stderror** | **tval** | **pval** |
| CA1 | 0.0027 | 0.0023 | 1.20 | 0.24 | 0.0032 | 0.0026 | 1.20 | 0.23 |
| CA3 | 0.0009 | 0.0015 | 0.61 | 0.54 | 0.0017 | 0.0013 | 1.30 | 0.20 |
| CA4 | -0.0002 | 0.0007 | -0.30 | 0.76 | 0.0008 | 0.0007 | 1.15 | 0.26 |
| subiculum | -0.0011 | 0.0017 | -0.62 | 0.53 | -0.0005 | 0.0016 | -0.33 | 0.74 |
| molecular_layer_HP | -0.0006 | 0.0009 | -0.61 | 0.54 | 0.0014 | 0.0009 | 1.66 | 0.10 |
| GC.ML.DG | -0.0003 | 0.0008 | -0.44 | 0.66 | 0.0008 | 0.0007 | 1.10 | 0.28 |

**Supplemental Table 6.** Descriptive statistics from linear mixed effect model investigating change in subfield volume throughout treatment.

|  | **Left** | | **Right** | |
| --- | --- | --- | --- | --- |
| **ROI** | **F** | **pval** | **F** | **pval** |
| CA1 | 0.15 | 0.70 | 0.51 | 0.47 |
| CA3 | 0.01 | 0.92 | 0.02 | 0.90 |
| CA4 | 0.01 | 0.94 | 0.04 | 0.85 |
| Subiculum | 0.74 | 0.39 | 0.02 | 0.88 |
| molecular_layer_HP | 0.07 | 0.79 | 0.05 | 0.82 |
| GC.ML.DG | 0.06 | 0.81 | 0.001 | 0.97 |

**Supplemental Table 7**. Descriptive statistics from linear regression investigating associations between pretreatment subfield volume and change in neurocognitive performance following single ketamine infusion (TP2). Associations that passed Bonferroni correction for multiple comparisons (0.008) are also marked with an asterisk.

|  |  | **LEFT** | | | | **RIGHT** | | | |
| --- | --- | --- | --- | --- | --- | --- | --- | --- | --- |
| **test** | **ROI** | **estimate** | **stderror** | **tval** | **pval** | **estimate** | **stderror** | **tval** | **pval** |
| fluid | CA1 | 3.16 | 2.84 | 1.11 | 0.27 | 2.47 | 2.46 | 1.00 | 0.32 |
| fluid | CA3 | 2.85 | 4.77 | 0.60 | 0.55 | 3.37 | 5.15 | 0.66 | 0.52 |
| fluid | CA4 | -3.72 | 9.27 | -0.40 | 0.69 | -5.50 | 10.40 | -0.53 | 0.60 |
| fluid | subiculum | -3.41 | 3.15 | -1.08 | 0.29 | -1.65 | 3.62 | -0.46 | 0.65 |
| fluid | molecular_layer_HP | -0.70 | 6.70 | -0.11 | 0.92 | -1.86 | 6.81 | -0.27 | 0.79 |
| fluid | GC.ML.DG | -5.03 | 7.79 | -0.65 | 0.52 | 6.17 | 9.33 | 0.66 | 0.51 |
| crystallized | CA1 | -0.98 | 1.97 | -0.50 | 0.62 | 0.49 | 1.72 | 0.28 | 0.78 |
| crystallized | CA3 | -0.27 | 3.28 | -0.08 | 0.94 | -0.77 | 3.57 | -0.22 | 0.83 |
| crystallized | CA4 | 4.02 | 6.32 | 0.64 | 0.53 | -4.41 | 7.17 | -0.61 | 0.54 |
| crystallized | subiculum | 0.39 | 2.19 | 0.18 | 0.86 | 1.07 | 2.50 | 0.43 | 0.67 |
| crystallized | molecular_layer_HP | 0.66 | 4.58 | 0.14 | 0.89 | 4.16 | 4.66 | 0.89 | 0.38 |
| crystallized | GC.ML.DG | 1.60 | 5.35 | 0.30 | 0.77 | -0.47 | 6.48 | -0.07 | 0.94 |
| flanker inhibition | CA1 | 3.34 | 3.33 | 1.00 | 0.32 | 1.47 | 2.90 | 0.51 | 0.62 |
| flanker inhibition | CA3 | 6.73 | 5.49 | 1.23 | 0.23 | 1.65 | 6.03 | 0.27 | 0.79 |
| flanker inhibition | CA4 | 5.01 | 10.82 | 0.46 | 0.65 | -4.90 | 12.14 | -0.40 | 0.69 |
| flanker inhibition | subiculum | -6.37 | 3.59 | -1.77 | 0.08 | -4.73 | 4.16 | -1.14 | 0.26 |
| flanker inhibition | molecular_layer_HP | -3.47 | 7.81 | -0.44 | 0.66 | -7.11 | 7.86 | -0.90 | 0.37 |
| flanker inhibition | GC.ML.DG | 5.02 | 9.12 | 0.55 | 0.59 | 6.45 | 10.88 | 0.59 | 0.56 |
| list sorting | CA1 | 1.30 | 3.73 | 0.35 | 0.73 | 3.11 | 3.19 | 0.98 | 0.34 |
| list sorting | CA3 | 11.98 | 5.89 | 2.03 | **0.05** | 13.90 | 6.32 | 2.20 | **0.03** |
| list sorting | CA4 | 19.20 | 11.61 | 1.65 | 0.11 | 5.71 | 13.49 | 0.42 | 0.67 |
| list sorting | subiculum | -6.65 | 4.00 | -1.67 | 0.10 | -5.60 | 4.62 | -1.21 | 0.23 |
| list sorting | molecular_layer_HP | 2.40 | 8.66 | 0.28 | 0.78 | 3.23 | 8.82 | 0.37 | 0.72 |
| list sorting | GC.ML.DG | 18.05 | 9.70 | 1.86 | 0.07 | 6.12 | 12.12 | 0.51 | 0.62 |
| cardsorting | CA1 | 2.09 | 3.95 | 0.53 | 0.60 | 1.35 | 3.29 | 0.41 | 0.68 |
| cardsorting | CA3 | 4.49 | 6.54 | 0.69 | 0.50 | 4.30 | 6.81 | 0.63 | 0.53 |
| cardsorting | CA4 | 3.85 | 12.74 | 0.30 | 0.76 | -3.22 | 13.79 | -0.23 | 0.82 |
| cardsorting | subiculum | -8.43 | 4.18 | -2.02 | **0.05** | -5.35 | 4.72 | -1.13 | 0.26 |
| cardsorting | molecular_layer_HP | -0.44 | 9.20 | -0.05 | 0.96 | 3.90 | 9.00 | 0.43 | 0.67 |
| cardsorting | GC.ML.DG | 4.07 | 10.74 | 0.38 | 0.71 | 7.65 | 12.34 | 0.62 | 0.54 |
| processing speed | CA1 | 9.56 | 4.59 | 2.08 | 0.04 | 6.69 | 4.02 | 1.66 | 0.10 |
| processing speed | CA3 | -21.46 | 7.25 | -2.96 | **0.01** | -19.63 | 8.04 | -2.44 | **0.02** |
| processing speed | CA4 | -52.51 | 13.05 | -4.02 | **0.0003*** | -38.21 | 16.29 | -2.34 | **0.02** |
| processing speed | subiculum | 11.40 | 5.04 | 2.26 | **0.03** | 8.42 | 5.91 | 1.43 | 0.16 |
| processing speed | molecular_layer_HP | 6.48 | 11.19 | 0.58 | 0.57 | 8.28 | 11.31 | 0.73 | 0.47 |
| processing speed | GC.ML.DG | -40.29 | 11.40 | -3.53 | **0.001*** | -21.44 | 15.28 | -1.40 | 0.17 |
| picture sequence | CA1 | -5.37 | 3.74 | -1.43 | 0.16 | -5.07 | 3.05 | -1.66 | 0.10 |
| picture sequence | CA3 | 9.59 | 6.19 | 1.55 | 0.13 | 6.35 | 6.48 | 0.98 | 0.33 |
| picture sequence | CA4 | 14.43 | 12.14 | 1.19 | 0.24 | 9.34 | 13.13 | 0.71 | 0.48 |
| picture sequence | subiculum | -1.45 | 4.25 | -0.34 | 0.73 | 1.53 | 4.59 | 0.33 | 0.74 |
| picture sequence | molecular_layer_HP | -4.30 | 8.89 | -0.48 | 0.63 | -15.48 | 8.26 | -1.87 | 0.07 |
| picture sequence | GC.ML.DG | 1.38 | 10.42 | 0.13 | 0.90 | 8.03 | 11.81 | 0.68 | 0.50 |
| oral reading | CA1 | 0.39 | 2.25 | 0.17 | 0.86 | 1.27 | 1.93 | 0.66 | 0.52 |
| oral reading | CA3 | -0.75 | 3.73 | -0.20 | 0.84 | -2.04 | 4.01 | -0.51 | 0.61 |
| oral reading | CA4 | 5.01 | 7.19 | 0.70 | 0.49 | 2.03 | 8.10 | 0.25 | 0.80 |
| oral reading | subiculum | 1.31 | 2.48 | 0.53 | 0.60 | 2.81 | 2.78 | 1.01 | 0.32 |
| oral reading | molecular_layer_HP | 0.11 | 5.22 | 0.02 | 0.98 | 3.72 | 5.26 | 0.71 | 0.48 |
| oral reading | GC.ML.DG | 4.14 | 6.07 | 0.68 | 0.50 | 5.27 | 7.24 | 0.73 | 0.47 |
| picture vocab | CA1 | -1.82 | 2.14 | -0.85 | 0.40 | -0.12 | 1.89 | -0.06 | 0.95 |
| picture vocab | CA3 | 0.45 | 3.58 | 0.13 | 0.90 | 0.67 | 3.92 | 0.17 | 0.87 |
| picture vocab | CA4 | 0.36 | 6.94 | 0.05 | 0.96 | -11.57 | 7.69 | -1.50 | 0.14 |
| picture vocab | subiculum | -0.14 | 2.39 | -0.06 | 0.95 | -0.04 | 2.75 | -0.02 | 0.99 |
| picture vocab | molecular_layer_HP | 0.85 | 5.00 | 0.17 | 0.87 | 4.05 | 5.13 | 0.79 | 0.43 |
| picture vocab | GC.ML.DG | -3.22 | 5.83 | -0.55 | 0.58 | -7.58 | 7.01 | -1.08 | 0.29 |

**Supplemental Table 8**. Descriptive statistics from linear regression investigating associations between pretreatment subfield volume and change in neurocognitive performance following serial ketamine infusion (TP3). Associations with p≤0.05 are indicated in bold. Associations that passed Bonferroni correction for multiple comparisons (0.008) are also marked with an asterisk.

|  |  | **LEFT** | | | | **RIGHT** | | | |
| --- | --- | --- | --- | --- | --- | --- | --- | --- | --- |
| **test** | **ROI** | **estimate** | **stderr** | **tval** | **pval** | **estimate** | **stderror** | **tval** | **pval** |
| fluid | CA1 | 2.04 | 2.05 | 0.99 | 0.32 | 1.64 | 1.89 | 0.87 | 0.39 |
| fluid | CA3 | 0.88 | 3.39 | 0.26 | 0.80 | 0.32 | 3.82 | 0.08 | 0.93 |
| fluid | CA4 | -1.24 | 7.36 | -0.17 | 0.87 | -1.36 | 7.35 | -0.19 | 0.85 |
| fluid | subiculum | -3.54 | 2.77 | -1.28 | 0.21 | -1.16 | 3.05 | -0.38 | 0.71 |
| fluid | molecular_layer_HP | 3.77 | 5.21 | 0.72 | 0.47 | 8.71 | 5.41 | 1.61 | 0.11 |
| fluid | GC.ML.DG | 0.22 | 6.25 | 0.03 | 0.97 | 4.39 | 6.49 | 0.68 | 0.50 |
| crystallized | CA1 | -1.02 | 1.37 | -0.74 | 0.46 | 0.27 | 1.30 | 0.21 | 0.84 |
| crystallized | CA3 | -1.44 | 2.25 | -0.64 | 0.52 | -2.17 | 2.60 | -0.83 | 0.41 |
| crystallized | CA4 | 2.82 | 4.89 | 0.58 | 0.57 | -3.91 | 5.01 | -0.78 | 0.44 |
| crystallized | subiculum | -0.10 | 1.87 | -0.06 | 0.96 | -0.06 | 2.10 | -0.03 | 0.98 |
| crystallized | molecular_layer_HP | -2.40 | 3.47 | -0.69 | 0.49 | 3.16 | 3.78 | 0.84 | 0.41 |
| crystallized | GC.ML.DG | 1.98 | 4.16 | 0.48 | 0.64 | -1.18 | 4.47 | -0.26 | 0.79 |
| flanker inhibition | CA1 | 1.86 | 2.70 | 0.69 | 0.49 | 0.15 | 2.47 | 0.06 | 0.95 |
| flanker inhibition | CA3 | 0.40 | 4.45 | 0.09 | 0.93 | -1.50 | 4.96 | -0.30 | 0.76 |
| flanker inhibition | CA4 | 5.58 | 9.63 | 0.58 | 0.56 | 4.25 | 9.53 | 0.45 | 0.66 |
| flankerinhibition | subiculum | -0.74 | 3.69 | -0.20 | 0.84 | 0.73 | 3.97 | 0.18 | 0.86 |
| flankerinhibition | molecular_layer_HP | 4.91 | 6.83 | 0.72 | 0.48 | 9.68 | 7.08 | 1.37 | 0.18 |
| flankerinhibition | GC.ML.DG | 9.80 | 8.09 | 1.21 | 0.23 | 9.57 | 8.36 | 1.14 | 0.26 |
| listsorting | CA1 | -1.39 | 3.33 | -0.42 | 0.68 | -0.32 | 3.04 | -0.10 | 0.92 |
| listsorting | CA3 | 13.64 | 5.13 | 2.66 | **0.01** | 13.48 | 5.81 | 2.32 | **0.02** |
| listsorting | CA4 | 27.12 | 11.24 | 2.41 | **0.02** | 16.43 | 11.53 | 1.43 | 0.16 |
| listsorting | subiculum | -8.91 | 4.36 | -2.05 | **0.05** | -9.11 | 4.72 | -1.93 | 0.06 |
| listsorting | molecular_layer_HP | 3.66 | 8.42 | 0.43 | 0.67 | 1.73 | 8.87 | 0.20 | 0.85 |
| listsorting | GC.ML.DG | 19.37 | 9.71 | 2.00 | **0.05** | 12.79 | 10.27 | 1.25 | 0.22 |
| cardsorting | CA1 | 6.27 | 2.70 | 2.33 | **0.02** | 5.09 | 2.48 | 2.06 | **0.04** |
| cardsorting | CA3 | 0.63 | 4.65 | 0.13 | 0.89 | -3.56 | 5.15 | -0.69 | 0.49 |
| cardsorting | CA4 | -1.85 | 10.08 | -0.18 | 0.86 | -7.86 | 9.90 | -0.79 | 0.43 |
| cardsorting | subiculum | -4.47 | 3.80 | -1.18 | 0.24 | 2.90 | 4.12 | 0.70 | 0.48 |
| cardsorting | molecular_layer_HP | 2.29 | 7.17 | 0.32 | 0.75 | 9.15 | 7.41 | 1.24 | 0.22 |
| cardsorting | GC.ML.DG | 3.83 | 8.55 | 0.45 | 0.66 | 0.04 | 8.83 | 0.00 | 1.00 |
| processingspeed | CA1 | 5.97 | 4.69 | 1.27 | 0.21 | 6.51 | 4.27 | 1.53 | 0.13 |
| processingspeed | CA3 | -19.75 | 7.31 | -2.70 | **0.01** | -16.59 | 8.45 | -1.96 | 0.06 |
| processingspeed | CA4 | -46.35 | 15.66 | -2.96 | **0.005** | -32.52 | 16.23 | -2.00 | **0.05** |
| processingspeed | subiculum | 14.15 | 6.16 | 2.30 | **0.03** | 6.65 | 6.95 | 0.96 | 0.34 |
| processingspeed | molecular_layer_HP | 10.75 | 11.96 | 0.90 | 0.37 | 14.87 | 12.55 | 1.18 | 0.24 |
| processingspeed | GC.ML.DG | -34.31 | 13.58 | -2.53 | **0.01** | -18.27 | 14.73 | -1.24 | 0.22 |
| picturesequence | CA1 | -5.19 | 3.78 | -1.37 | 0.18 | -4.01 | 3.50 | -1.15 | 0.26 |
| picturesequence | CA3 | 1.42 | 6.31 | 0.22 | 0.82 | 3.30 | 7.10 | 0.47 | 0.64 |
| picturesequence | CA4 | 0.28 | 13.69 | 0.02 | 0.98 | 1.44 | 13.68 | 0.11 | 0.92 |
| picturesequence | subiculum | -5.17 | 5.18 | -1.00 | 0.32 | -4.27 | 5.66 | -0.75 | 0.45 |
| picturesequence | molecular_layer_HP | -5.95 | 9.70 | -0.61 | 0.54 | -2.25 | 10.33 | -0.22 | 0.83 |
| picturesequence | GC.ML.DG | -2.20 | 11.63 | -0.19 | 0.85 | -0.35 | 12.14 | -0.03 | 0.98 |
| oralreading | CA1 | -1.65 | 1.88 | -0.88 | 0.38 | 0.28 | 1.88 | 0.15 | 0.88 |
| oralreading | CA3 | -4.50 | 3.04 | -1.48 | 0.14 | -2.08 | 3.77 | -0.55 | 0.58 |
| oralreading | CA4 | 0.45 | 6.73 | 0.07 | 0.95 | -0.42 | 7.28 | -0.06 | 0.95 |
| oralreading | subiculum | 3.47 | 2.52 | 1.37 | 0.18 | -1.25 | 3.03 | -0.41 | 0.68 |
| oralreading | molecular_layer_HP | -1.36 | 4.78 | -0.29 | 0.78 | 4.73 | 5.46 | 0.87 | 0.39 |
| oralreading | GC.ML.DG | 1.43 | 5.71 | 0.25 | 0.80 | 0.96 | 6.46 | 0.15 | 0.88 |
| picturevocab | CA1 | -0.07 | 1.90 | -0.03 | 0.97 | 0.10 | 1.78 | 0.06 | 0.95 |
| picturevocab | CA3 | 2.99 | 3.09 | 0.97 | 0.34 | -1.84 | 3.57 | -0.52 | 0.61 |
| picturevocab | CA4 | 4.83 | 6.72 | 0.72 | 0.48 | -6.62 | 6.83 | -0.97 | 0.34 |
| picturevocab | subiculum | -4.09 | 2.52 | -1.63 | 0.11 | 2.70 | 2.84 | 0.95 | 0.35 |
| picturevocab | molecular_layer_HP | -4.15 | 4.77 | -0.87 | 0.39 | 0.39 | 5.20 | 0.08 | 0.94 |
| picturevocab | GC.ML.DG | 2.11 | 5.73 | 0.37 | 0.71 | -3.90 | 6.09 | -0.64 | 0.52 |

**Supplemental Table 9**. Descriptive statistics from linear regression investigating associations between pretreatment subfield volume and change in neurocognitive performance at follow-up (TP4). Associations with p≤0.05 are indicated in bold. Associations that passed Bonferroni correction for multiple comparisons (0.008) are also marked with an asterisk.

|  |  | **LEFT** | | | | **RIGHT** | | | |
| --- | --- | --- | --- | --- | --- | --- | --- | --- | --- |
| **test** | **ROI** | **estimate** | **stderror** | **tval** | **pval** | **estimate** | **stderror** | **tval** | **pval** |
| fluid | CA1 | 0.60 | 2.97 | 0.20 | 0.84 | -3.76 | 2.51 | -1.50 | 0.15 |
| fluid | CA3 | 3.18 | 5.21 | 0.61 | 0.55 | 3.81 | 6.81 | 0.56 | 0.58 |
| fluid | CA4 | 2.67 | 10.77 | 0.25 | 0.81 | 8.62 | 10.64 | 0.81 | 0.42 |
| fluid | subiculum | -7.07 | 5.30 | -1.33 | 0.19 | -3.50 | 4.40 | -0.79 | 0.43 |
| fluid | molecular_layer_HP | 3.82 | 7.80 | 0.49 | 0.63 | -7.44 | 8.22 | -0.91 | 0.37 |
| fluid | GC.ML.DG | 3.43 | 8.82 | 0.39 | 0.70 | 11.68 | 10.12 | 1.15 | 0.26 |
| crystallized | CA1 | -1.07 | 2.13 | -0.50 | 0.62 | 0.74 | 1.88 | 0.40 | 0.70 |
| crystallized | CA3 | 0.83 | 3.78 | 0.22 | 0.83 | -1.29 | 4.92 | -0.26 | 0.79 |
| crystallized | CA4 | 9.20 | 7.54 | 1.22 | 0.23 | 3.21 | 7.73 | 0.42 | 0.68 |
| crystallized | subiculum | 1.45 | 3.93 | 0.37 | 0.72 | -0.46 | 3.21 | -0.14 | 0.89 |
| crystallized | molecular_layer_HP | -2.49 | 5.62 | -0.44 | 0.66 | -0.42 | 6.01 | -0.07 | 0.94 |
| crystallized | GC.ML.DG | 6.48 | 6.23 | 1.04 | 0.31 | 3.46 | 7.44 | 0.47 | 0.65 |
| flankerinhibition | CA1 | 0.71 | 3.75 | 0.19 | 0.85 | -4.31 | 3.21 | -1.34 | 0.19 |
| flankerinhibition | CA3 | -1.97 | 6.63 | -0.30 | 0.77 | -5.90 | 8.63 | -0.68 | 0.50 |
| flankerinhibition | CA4 | 9.00 | 13.52 | 0.67 | 0.51 | 13.32 | 13.44 | 0.99 | 0.33 |
| flankerinhibition | subiculum | 0.94 | 6.93 | 0.14 | 0.89 | -0.68 | 5.66 | -0.12 | 0.90 |
| flankerinhibition | molecular_layer_HP | 6.76 | 9.82 | 0.69 | 0.50 | -3.53 | 10.59 | -0.33 | 0.74 |
| flankerinhibition | GC.ML.DG | 11.71 | 10.94 | 1.07 | 0.29 | 11.87 | 12.98 | 0.91 | 0.37 |
| listsorting | CA1 | 0.74 | 5.29 | 0.14 | 0.89 | 2.44 | 4.70 | 0.52 | 0.61 |
| listsorting | CA3 | 22.20 | 8.24 | 2.69 | **0.01** | 37.74 | 9.92 | 3.80 | **0.0007*** |
| listsorting | CA4 | 42.02 | 17.28 | 2.43 | **0.02** | 34.78 | 18.24 | 1.91 | 0.07 |
| listsorting | subiculum | -16.49 | 9.20 | -1.79 | 0.09 | -16.93 | 7.34 | -2.31 | **0.03** |
| listsorting | molecular_layer_HP | 8.72 | 13.86 | 0.63 | 0.54 | 18.19 | 14.66 | 1.24 | 0.23 |
| listsorting | GC.ML.DG | 35.82 | 14.05 | 2.55 | **0.02** | 36.45 | 17.34 | 2.10 | **0.05** |
| cardsorting | CA1 | -0.91 | 4.77 | -0.19 | 0.85 | -4.02 | 4.16 | -0.97 | 0.34 |
| cardsorting | CA3 | 2.31 | 8.42 | 0.27 | 0.79 | 5.92 | 11.03 | 0.54 | 0.60 |
| cardsorting | CA4 | 16.75 | 16.99 | 0.99 | 0.33 | 12.83 | 17.26 | 0.74 | 0.46 |
| cardsorting | subiculum | -12.84 | 8.42 | -1.52 | 0.14 | -4.66 | 7.16 | -0.65 | 0.52 |
| cardsorting | molecular_layer_HP | -1.94 | 12.58 | -0.15 | 0.88 | -8.78 | 13.41 | -0.65 | 0.52 |
| cardsorting | GC.ML.DG | 14.35 | 13.92 | 1.03 | 0.31 | 16.46 | 16.49 | 1.00 | 0.33 |
| processingspeed | CA1 | 1.16 | 4.76 | 0.24 | 0.81 | 0.74 | 4.20 | 0.18 | 0.86 |
| processingspeed | CA3 | -10.58 | 8.15 | -1.30 | 0.21 | -24.71 | 9.87 | -2.50 | **0.02** |
| processingspeed | CA4 | -39.15 | 15.41 | -2.54 | **0.02** | -37.32 | 15.67 | -2.38 | **0.02** |
| processingspeed | subiculum | 9.43 | 8.59 | 1.10 | 0.28 | 8.53 | 6.96 | 1.23 | 0.23 |
| processingspeed | molecular_layer_HP | 2.01 | 12.56 | 0.16 | 0.87 | -2.33 | 13.40 | -0.17 | 0.86 |
| processingspeed | GC.ML.DG | -35.09 | 12.32 | -2.85 | **0.01** | -35.30 | 15.16 | -2.33 | **0.03** |
| picturesequence | CA1 | -0.55 | 4.79 | -0.12 | 0.91 | -5.07 | 4.13 | -1.23 | 0.23 |
| picturesequence | CA3 | 0.80 | 8.48 | 0.09 | 0.93 | -1.35 | 11.12 | -0.12 | 0.90 |
| picturesequence | CA4 | -16.55 | 17.09 | -0.97 | 0.34 | -4.94 | 17.47 | -0.28 | 0.78 |
| picturesequence | subiculum | -4.73 | 8.80 | -0.54 | 0.60 | 2.44 | 7.23 | 0.34 | 0.74 |
| picturesequence | molecular_layer_HP | -1.09 | 12.65 | -0.09 | 0.93 | -20.71 | 12.94 | -1.60 | 0.12 |
| picturesequence | GC.ML.DG | -13.10 | 14.04 | -0.93 | 0.36 | 0.25 | 16.86 | 0.01 | 0.99 |
| oralreading | CA1 | -1.76 | 2.87 | -0.62 | 0.54 | -1.08 | 2.54 | -0.42 | 0.67 |
| oralreading | CA3 | -2.20 | 5.09 | -0.43 | 0.67 | -6.36 | 6.55 | -0.97 | 0.34 |
| oralreading | CA4 | 9.73 | 10.30 | 0.94 | 0.35 | 7.01 | 10.39 | 0.67 | 0.51 |
| oralreading | subiculum | 8.01 | 5.08 | 1.58 | 0.13 | 1.69 | 4.33 | 0.39 | 0.70 |
| oralreading | molecular_layer_HP | -1.50 | 7.61 | -0.20 | 0.84 | -7.49 | 7.99 | -0.94 | 0.36 |
| oralreading | GC.ML.DG | 8.53 | 8.43 | 1.01 | 0.32 | 6.67 | 10.01 | 0.67 | 0.51 |
| picturevocab | CA1 | 0.02 | 2.52 | 0.01 | 0.99 | 2.64 | 2.16 | 1.23 | 0.23 |
| picturevocab | CA3 | 5.42 | 4.32 | 1.25 | 0.22 | 7.27 | 5.63 | 1.29 | 0.21 |
| picturevocab | CA4 | 6.68 | 9.05 | 0.74 | 0.47 | -0.44 | 9.13 | -0.05 | 0.96 |
| picturevocab | subiculum | -5.68 | 4.51 | -1.26 | 0.22 | -3.13 | 3.73 | -0.84 | 0.41 |
| picturevocab | molecular_layer_HP | -4.25 | 6.59 | -0.64 | 0.52 | 6.10 | 6.98 | 0.87 | 0.39 |
| picturevocab | GC.ML.DG | 2.20 | 7.49 | 0.29 | 0.77 | -1.04 | 8.80 | -0.12 | 0.91 |

**Baseline hippocampal volume in healthy controls and practice effects.**

We performed a supplementary analysis testing for associations between baseline healthy control hippocampal volumes and change in neurocognitive performance in a subset of N=17 healthy controls (age=28.2±6.8, sex=8M/9F). NIHToolbox measures were acquired at baseline and at a 2-week follow-up (a similar interval to patients assessed pre and post ketamine). No significant associations between baseline hippocampal subfield volume and change in neurocognitive performance were observed in controls in either hemisphere.
